# Insights for the treatment of depression in the Brazilian Public Health System

**DOI:** 10.1590/1516-3180.2025.3068.14052025

**Published:** 2025-10-27

**Authors:** Thales Marcon Almeida, Ana Lídia Marcon Almeida, Quirino Cordeiro, Ricardo Riyoiti Uchida

**Affiliations:** I Professor, Faculdade de Ciências Médicas, Santa Casa de São Paulo, São Paulo (SP), Brazil.; II Physician, Pontifícia Universidade Católica de São Paulo, Sorocaba (SP), Brazil; III Professor, Faculdade de Ciências Médicas, Santa Casa de São Paulo, São Paulo (SP), Brazil.; IV Professor, Faculdade de Ciências Médicas, Santa Casa de São Paulo, São Paulo (SP), Brazil.

Dear Editor,

 A recent article by Cleare et al.^1^ provides valuable insights into the pharmacological management of treatment-resistant depression (TRD), particularly regarding the use of adjunctive agents. Over a 52-week follow-up period, the study found that participants treated with adjunctive quetiapine exhibited significantly lower depressive symptom severity, reduced healthcare costs, greater gains in Quality-Adjusted Life Years (QALYs), and no differences in discontinuation rates compared to those receiving lithium. These findings raise important considerations for the treatment of depressive episodes within the Brazilian Public Health System (Sistema Único de Saúde, SUS), especially in cases of TRD. 

 Taking the state of São Paulo as an example, which is the most populous in Brazil, with over 44 million inhabitants, representing approximately 20% of the country’s population, the estimated prevalence of depression in primary care is around 25%.^2^ Most of these patients receive treatment through the public health system, where certain medications are provided free of charge with a medical prescription. Available treatments for depressive episodes include selective serotonin reuptake inhibitors (fluoxetine and sertraline), tricyclic antidepressants (clomipramine, amitriptyline, nortriptyline, and imipramine), and, in some regions, the serotonin and norepinephrine reuptake inhibitor venlafaxine. 

 Regarding evidence-based adjunctive strategies,^3^ only lithium, risperidone, and immediate-release methylphenidate are currently available. Quetiapine, along with other atypical antipsychotics, is included in the formulary solely for patients diagnosed with bipolar disorder or schizophrenia, consequently making its use in major depressive disorder inaccessible through the public system. Furthermore, given Brazil’s socioeconomic landscape, many patients cannot afford the monthly cost of private prescriptions. This is particularly relevant considering that among primary care users, psychiatric diagnoses are more prevalent in individuals with lower income, unemployment, and limited access to education.^2^

 In Latin America, Brazil has the highest prevalence of TRD, ultimately affecting nearly 40% of patients with depression, with higher rates observed in public healthcare settings compared to private services.^4^ In addition, compared to non-resistant depressive episodes, TRD in Brazil imposes significantly greater costs on the public health system, thus requiring increased resource allocation, including higher hospitalization rates and multiple pharmacological trials, with pharmaceutical expenditures representing the largest proportion of total costs.^5^

 Given this scenario and considering that quetiapine is among the most effective adjunctive treatments for TRD,^6^ for mental health professionals, particularly those working in the public sector, to advocate for expanding treatment options is crucial. Doing so could improve response and remission rates, enhance patient quality of life, and potentially reduce the long-term financial burden on the healthcare system. 
